# Assessment of quality of life in people with severe and enduring anorexia nervosa: a comparison of generic and specific instruments

**DOI:** 10.1186/2050-2974-1-S1-O42

**Published:** 2013-11-14

**Authors:** Deborah Mitchison, Phillipa Hay, Scott Engel, Ross Crosby, Daniel Le Grange, Hubert Lacey, Jonathan Mond, Shameran Slewa-Younan, Stephen Touyz

**Affiliations:** 1 School of Medicine, University of Western Sydney, Australia; 2 Centre for Health Research, School of Medicine, University of Western Sydney, Australia; 3 School of Medicine, James Cook University, Australia; 4 Neuropsychiatric Research Institute, Fargo, School of Medicine and Health Sciences, University of North Dakota, USA; 5 Department of Psychiatry and Behavioral Neuroscience, University of Chicago, USA; 6 Division of Population Health Sciences and Education, St George's, University of London, UK; 7 School of Medicine and Public Health, University of Newcastle, Australia; 8 School of Sociology, Australian National University, Australia; 9 School of Medicine, University of Western Sydney, Australia; 10 School of Psychology, University of Sydney, Australia

## Aims

To compare the psychometric properties of generic and disease-specific measures of health-related quality of life (HRQoL).

## Methods

63 participants with anorexia nervosa (AN) completed measures of generic HRQoL (Medical Outcomes Study Short-Form; SF-12), disease-specific HRQoL (Eating Disorders Quality of Life Questionnaire; EDQOL), functional impairment (days out of role; Work and Social Adjustment Scale), and eating disorder severity (Eating Disorder Examination Questionnaire) at baseline, post-treatment, and 6-12 months follow-up. The SF-12 and EDQOL were compared on internal consistency, convergence validity, and criterion-related validity.

## Results

The EDQOL had stronger internal consistency α = 0.92 vs. 0.80), convergence with eating disorder severity (r = 0.06 to 0.53 vs. 0.11 to -0.39), and criterion-related (functional impairment) validity (β = 0.37 - 0.46) compared to the SF-12. The SF-12 converged more strongly with functional impairment (r = -0.31 to -0.63 vs. 0.06 to 0.70). The SF-12 (β = 0.41 - 0.42) and EDQOL (β = 0.33 - 0.38) had similar criterion-related (eating disorder severity) validity.

## Conclusions

The EDQOL and SF-12 should be used where possible in tandem as measures of HRQoL, whereas the SF-12 may be preferable in studies where comparisons are to be made with other populations.

This abstract was presented in the **Anorexia Nervosa – Characteristics and Treatment** stream of the 2013 ANZAED Conference.

